# Work environment, volume of activity and staffing in neonatal intensive care units in Italy: results of the SONAR-nurse study

**DOI:** 10.1186/s13052-016-0247-6

**Published:** 2016-04-02

**Authors:** Carlo Corchia, Simone Fanelli, Luigi Gagliardi, Roberto Bellù, Antonello Zangrandi, Anna Persico, Rinaldo Zanini, on behalf of the SONAR-Nurse Study Group

**Affiliations:** ICBD, Alessandra Lisi International Centre on Birth Defects and Prematurity, Rome, Italy; Department of Economics, Parma University, Parma, Italy; Woman and Child Health Department, Ospedale Versilia, Viareggio, Italy; Neonatal Intensive Care Unit, Alessandro Manzoni Hospital, Lecco, Italy; Neonatal Unit, University of Turin, Turin, Italy; Woman and Child Health Department, Azienda Ospedaliera Province of Lecco, Lecco, Italy

**Keywords:** Infant, Newborn, Intensive Care Units, Neonatal, Nurse-Patient Relations, Patient Acuity, Workload

## Abstract

**Background:**

Neonatal units’ volume of activity, and other quantitative and qualitative variables, such as staffing, workload, work environment, care organization and geographical location, may influence the outcome of high risk newborns. Data about the distribution of these variables and their relationships among Italian neonatal units are lacking.

**Methods:**

Between March 2010-April 2011, 63 neonatal intensive care units adhering to the Italian Neonatal Network participated in the SONAR Nurse study. Their main features and work environment were investigated by questionnaires compiled by the chief and by physicians and nurses of each unit. Twelve cross-sectional monthly-repeated surveys on different shifts were performed, collecting data on number of nurses on duty and number and acuity of hospitalized infants.

**Results:**

Six hundred forty five physicians and 1601 nurses compiled the questionnaires. In the cross-sectional surveys 702 reports were collected, with 11082 infant and 3226 nurse data points. A high variability was found for units’ size (4–50 total beds), daily number of patients (median 14.5, range 3.4-48.7), number of nurses per shift (median 4.2, range 0.7-10.8) and number of team meetings per month. Northern regions performed better than Central and Southern regions for frequency of training meetings, qualitative assessment of performance, motivation within the unit and nursing work environment; mean physicians’ and nurses’ age increased moving from North to South. After stratification by terciles of the mean daily number of patients, the median number of nurses per shift increased at increasing volume of activity, while the opposite was found for the nurse-to-patient ratio adjusted by patients’ acuity. On average, in units belonging to the lower tercile there was 1 nurse every 2.5 patients, while in those belonging to the higher tercile the ratio was 1 nurse every 5 patients.

**Conclusions:**

In Italy, there is a high variability in organizational characteristics and work environment among neonatal units and an uneven distribution of human resources in relation to volume of activity, suggesting that the larger the unit the greater the workload for each nurse. Urgent modifications in planning and organization of services are needed in order to pursue more efficient, homogeneous and integrated regionalized neonatal care systems.

## Background

Differences in outcome of very preterm or very low birth weight (VLBW) infants and in characteristics of neonatal intensive care units (NICUs) may be present between different countries and also within the same country [1–5]. Various attempts tried to explain such disparities, but results were often inconsistent and sometimes contradictory. Level and size of birth hospital, volume of activity (frequently estimated by the number of high risk babies admitted to a single NICU during one calendar year), and the nurse workload, often measured by the nurse-to-patient ratio (NPR), were the most frequently studied variables.

The association between a worse outcome of very preterm or VLBW infants and to be born outside hospitals with the most specialized level of care has been definitively demonstrated [6]. On the contrary, the observation that high volumes of activity were associated with lower mortality [7–11] was not always confirmed [12, 13], suggesting that outcome can be influenced by other concurrent variables, such as those related to work contexts and care organization. Moreover, only a small fraction of the variability in mortality among different centers (9–15 %) was found to be explained by differences in volumes of activity after adjustment for acuity or case-mix of patients [4, 11]; at the same time a large proportion of low-volume NICUs can perform better than expected [11].

Similarly, when nurse workload and NPR were studied, a higher mortality was observed in association with a higher nurse workload in some contexts and the reverse in others, so preventing definite conclusions about optimal staffing [14]. The importance of some qualitative aspects of staffing, such as a positive effect of nurses’ neonatal qualification on mortality, emerged in some studies [15].

Although not frequently studied, also a better work environment has been found to be connected to better quality of care and infants’ safety and outcomes [16], especially when organizational factors facilitate the way in which care providers work together [17].

In Italy, a very wide variation in the number of admissions of VLBW infants among NICUs during one calendar year was found, along with striking geographical differences in mortality between Northern and Southern regions [18]. In adjunct, the NICUs’ average daily number of high-dependent infants appeared a better explanatory variable of outcome than the volume of activity [5], underscoring the importance of taking into account the care needs and the acuity of patients when comparing different care settings. More recently it has been also found that on average the Italian NICUs are relatively understaffed and that an inefficient mismatch is present between infants’ acuity and ward of care [19].

In the present study we aimed at: a) describing, in a group of Italian NICUs, the work environment, as experienced by physicians and nurses, the mean volume of activity and the mean NPR adjusted for the acuity of patients (aNPR), b) finding out whether differences exist among geographical areas and c) analyzing the relationships between the volume of activity and the other variables under investigation. Data collected in the SONAR Nurse survey were used.

## Methods

Between March 2010 and April 2011, 63 NICUs, representing 52 % of all those present in Italy and adhering to the Italian Neonatal Network (INN), a voluntary collaboration of Italian neonatal units - branch of the Vermont-Oxford Network -, participated in SONAR Nurse study.

### Data collection

In the first part of the survey, the chief of each NICUs compiled a questionnaire describing the main features, including administrative characteristics, of the unit. Two other questionnaires exploring the work environment within the unit were compiled by physicians and nurses on duty at each unit, respectively. The nurses’ questionnaire included the 31 items of the Practice Environment Scale – Nursing Work Index (PES-NWI), developed to measure the hospital nursing environment [20]. Written consent to participate in the study was given by physicians and nurses in each NICU.

In the second part of the study, 12 cross-sectional observational surveys were carried out monthly on different shifts (morning, afternoon, night, and holiday). Data on number of nurses and number and acuity of infants present were collected. At each survey, the nurses assessed the infants’ acuity using a modified Rogowski’s classification [21], in which the 5 original categories were ordered to represent a decreasing complexity: 1 = unstable, requiring complex critical care; 2 = multisystem support; 3 = intensive care; 4 = intermediate care; 5 = continuing care. This classification details the American Academy of Pediatrics/American College of Obstetricians and Gynecologists (AAP/ACOG) classification. [21, 22]. Other details about this classification and data collection were reported elsewhere [19]. Infants could be assessed in more than one repeated survey in case of long hospitalizations; “infant data points” rather than infants were therefore analyzed.

In order to make classification criteria as uniform as possible and to reduce variability, meetings with physicians’ and nurses’ staff leaders at each participating NICU were organized before the beginning of the survey. The study was performed in compliance with the Helsinki Declaration on medical research involving human subjects, and was approved by the Ethics Committee of Azienda Ospedaliera “Ospedale di Lecco” on March 04, 2009, ref. 140109. Local Ethics Committees’ approval was also sought by all units participating in the study.

Among the variables taken into account, 5 derived from the questionnaire about the characteristics of the NICUs: total number of beds in the hospital where the unit was located, total number of beds in each unit, geographical area, and age of physicians and nurses on duty in the NICU; 7 came from the physician questionnaire: number of team meetings per month (on clinical cases, about organizational aspects, and training sessions), judgment about the qualitative evaluation of performances, overall judgment about the presence of critical organizational issues (average of eight sub-scales), organization capacity to promote professionalism and competence, and motivation within the NICU (the original questionnaire is available on request); 1 represented the PES-NWI; and 4 derived from the monthly surveys: number of infants and nurses present at surveys, acuity score (AS), and aNPR. For ordinal variables numerical ranks were used.

### Statistical analysis

The analysis was carried out in two stages. Firstly, for each unit the means of the variables under study were calculated. The mean number of patients and nurses and the mean AS per day were computed summing up all the information collected in the monthly surveys and dividing the results by the number of surveys. The mean daily number of patients has been taken as a measure of the volume of activity of the units. The mean aNPR for each unit was calculated dividing the mean daily number of nurses by the adjusted mean daily number of patients; this last measure was obtained multiplying the observed mean daily number of patients by the ratio between the expected mean AS (i.e. the mean AS of the whole set of patients’ observations) and the observed mean AS. In this way the aNPR can be considered a measure of the nurse workload: the higher the aNPR, the lower the nurse workload.

As a second stage, the medians and ranges of the mean values so obtained for the 63 NICUs were computed, overall and stratifying by geographical area (Northern, Central and Southern regions) and by terciles of the mean number of patients per day.

Statistical analyses were performed with simple linear regression and the Kruskall-Wallis test using the Stata 11 package; [23] differences were considered statistically significant when *P* values were <0.05.

## Results

Of the 63 NICUs participating in the study, 31 were located in Northern regions (49 %), 10 in Central regions (16 %), and 22 in Southern regions (35 %); the overall percentages of liveborn infants in the three areas were 46 %, 20 % and 34 %, respectively in 2014 [24]. Six hundred forty five physicians and 1601 nurses compiled the questionnaires about the work environment.

In the cross-sectional surveys 702 reports were collected; forty five NICUs (71.4 %) compiled the questionnaire for all the 12 scheduled monthly surveys; for the other 18 NICUs the number of observations ranged between 11 and 4. Overall, data about 11082 infants and 3226 nurses were obtained.

The median values and ranges of the distribution of the variables under study among the 63 NICUs, in total and after stratification by geographical area, are shown in Table 1. The size of hospitals where the NICUs were located and the size of NICUs themselves were very variable, ranging between 140-1758, and 4–50 total beds, respectively. A high variability was also found for the number of meetings per month, in particular for meetings on clinical cases, whose average frequency was much higher than that of organizational and training meetings. The median daily number of patients was 14.5, with a very wide distribution of values, ranging between 3.4 and 48.7. The median number of nurses on duty per shift was 4.2, and also for this variable the distribution of values was very wide. Some differences were found among geographical areas. In particular, hospital sizes were highest in Northern and lowest in Southern regions, with intermediate value in Central regions; physicians’ and nurses’ age increased moving from North to South. Finally, Northern regions performed better than Central and Southern regions for training meetings, qualitative evaluation of performance, motivation within the unit and nursing work environment.

**Table 1:** Medians (ranges) of the distribution of the mean values of the variables under study for 63 Italian Neonatal Intensive Care Units, overall and stratified by geographical area

Variables	All NICUs	North (*n* = 31)	Centre (*n* = 10)	South (*n* = 22)	*P* value*
Hospital total beds, n.	669 (140–1758)	754 (211–1785)	544 (225–1348)	501.5 (140–1320)	0.0023
NICU total beds, n.	16 (4–50)	16 (4–50)	16 (8–37)	16 (6–31)	0.9907
Physicians’ age, years	44.3 (34–51.7)	41.8 (35.2-49.7)	44.3 (38.2-47.0)	47.7 (34–51.7)	0.003
Nurses’ age, years	38.8 (30.4-50.8)	37.1 (30.4-43.2)	38.8 (34.7-47.6)	42.9 (36.5-50.8)	0.0001
Team meetings on clinical cases, n. per month	3.6 (0.1-22.7)	3.6 (0.4-22.7)	2.2 (0.1-10.5)	3.8 (0.7-13.7)	0.5278
Team meetings about organizational aspects, n. per month	0.7 (0.03-3.9)	0.7 (0.2-3.9)	0.5 (0.1-3.8)	0.5 (0.03-2.9)	0.1088
Team training meetings, n. per month	0.5 (0.04-3.5)	0.8 (0.1-3.5)	0.4 (0.1-1.7)	0.4 (0.04-2.4)	0.0047
Judgment about the qualitative assessment of performances (1 worst; 5 best)	2.6 (1–4)	2.9 (1.3-4.0)	2.3 (1.0-3.2)	2.1 (1.0-3.0)	0.0004
Judgment about the presence of critical organizational issues (1 min; 10 max)	5.6 (2.6-7.7)	5.5 (2.6-7.5)	6.1 (3.9-7.7)	5.7 (4.7-7.3)	0.4782
Organization capacity to promote physicians’ professionalism and competence (1 always; 5 never)	2.4 (1.7-3.7)	2.1 (1.9-3.7)	2.6 (1.7-3.2)	2.4 (1.1-3.4)	0.4726
Motivation within the neonatal unit (1 highest; 5 lowest)	1.9 (1.0-3.0)	1.8 (1.3-2.7)	2.1 (1.4-2.5)	2.1 (1.0-3.0)	0.0338
Nursing work environment (1 worst; 5 best)	2.4 (1.9-3.0)	2.5 (2.1-3.0)	2.3 (2.0-2.8)	2.4 (1.9-2.8)	0.0424
Daily number of patients	14.5 (3.4-48.7)	15.5 (5.2-48.7)	14.8 (5.7-26.5)	14.3 (3.4-23.1)	0.7310
Acuity score	4.1 (3.0-4.6)	4.1 (0.7-10.8)	4.1 (2.5-8.2)	4.5 (1.8-6.6)	0.8456
Number of nurses on duty per shift	4.2 (0.7-10.8)	4.1 (3.7-4.6)	4.1 (3.4-4.5)	4.1 (3.0-4.6)	0.8343
Adjusted nurse-to-patient ratio	0.3 (0.1-0.9)	0.3 (0.1-0.8)	0.3 (0.2-0.6)	0.3 (0.2-0.9)	0.1518

*Differences between geographical areas

Some differences were found when NICUs were stratified by terciles of the mean daily number of patients (Table 2). In particular, hospital and NICU sizes increased at increasing terciles, while the contrary was observed for mean physicians’ age, that resulted lower in units belonging to the 1^st^ tercile. Although the median number of nurses on duty per shift increased at increasing volume of activity, for the aNPR the opposite was found. These results indicate that at high volumes of activity the increase in number of nurses was not such as to keep unchanged the nurse-to-patient ratio adjusted for patients’ AS; actually the ratio got worse. On average, in NICUs belonging to the 1^st^ tercile there was 1 nurse every 2.5 patients, while in NICUs belonging to the 3^rd^ tercile the ratio was 1 nurse every 5 patients.

**Table 2:** Medians (ranges) of the distribution of the mean values of the variables under study for 63 Italian Neonatal Intensive Care Units, overall and stratified by terciles of the mean daily number of patients

	Mean daily number of patients			
Variables	1^st^ tercile (<12.4 patients/day)	2^nd^ tercile (12.4-17.2 patients/day)	3^rd^ tercile (>17.2 patients/day)	*P* value
Hospital total beds, n.	505 (140–1368)	707 (276–1500)	903 (318–1758)	0.0029
NICU total beds, n.	12 (6–15)	16 (4–28)	24 (7–50)	0.0001
Physicians’ age, years	40.9 (34.0-48.2)	47.1 (36.2-51.7)	46.4 (39.9-50.9)	0.0021
Nurses’ age, years	40.9 (33.8-47.6)	39.2 (31.9-49.1)	37.3 (30.4-50.8)	0.0913
Team meetings on clinical cases, n. per month	2.1 (0.4-22.7)	6.0 (1.0-13.7)	3.0 (0.1-13.7)	0.2334
Team meetings about organizational aspects, n. per month	0.7 (0.1-3.1)	0.6 (0.03-3.9)	0.7 (0.3-3.8)	0.7064
Team training meetings, n. per month	0.4 (0.04-3.8)	0.6 (0.1-3.4)	0.5 (0.1-3.1)	0.3169
Judgment about the qualitative assessment of performances (1 worst; 5 best)	2.2 (1.0-4.0)	2.7 (1.2-3.5)	2.6 (1.3-3.7)	0.6337
Judgment about the presence of critical organizational issues (1 min; 10 max)	5.2 (3.9-7.3)	5.6 (2.6-7.3)	6.0 (2.8-7.7)	0.2580
Organization capacity to promote physicians’ professionalism and competence (1 always; 5 never)	2.3 (1.7-3.2)	2.4 (1.8-3.4)	2.5 (1.9-3.7)	0.3784
Motivation within the neonatal unit (1 highest; 5 lowest)	2.0 (1.0-2.7)	1.9 (1.3-3.0)	1.9 (1.5-2.6)	0.4541
Nursing work environment (1 worst; 5 best)	2.4 (1.9-2.8)	2.5 (2.1-2.8)	2.4 (2.0-3.0)	0.4611
Number of nurses on duty per shift	3.3 (0.7-5.6)	4.0 (2.8-5.0)	5.2 (4.0-10.8)	0.0001
Acuity score	4.1 (3.0-4.6)	4.2 (3.9-4.6)	4.1 (3.4-4.5)	0.1997
Adjusted nurse-to-patient ratio	0.4 (0.1-0.9)	0.3 (0.2-0.3)	0.2 (0.2-0.4)	0.0004

**Fig. 1:**
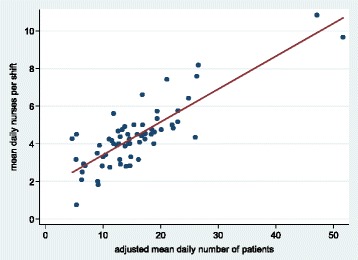
Scatterplot of mean daily nurses per shift against adjusted mean daily number of patients, with regression line (regression equation: mean daily nurses per shift = 1.6621 + 0.1756 x adjusted mean daily number of patients; *P* = 0.0000; *R* ^2^ = 0.70)

A statistically significant positive linear relationship was found between the adjusted mean daily number of patients and the mean daily number of nurses per shift (Fig. 1). On average, from the regression equation the estimated mean daily numbers of nurses per shift for 10, 20 and 30 mean daily numbers of patients were 2.8 (1 nurse every 3.5 infants), 5.2 (1 nurse every 3.9 infants) and 6.9 (1 nurse every 4.3 infants). Once again these results demonstrate that the increase in number of nurses was not such as to compensate for the increase in number of patients adjusted for AS.

## Discussion

When investigating the reasons for differences in outcome of neonatal intensive care, some easily collected and quantifiable explanatory variables such as the volume of activity and the NPR were found to explain only a very small fraction of the variability among NICUs, also when the characteristics and acuity of patients were taken into account [4–11]. Some other qualitative variables related to staff composition and work environment within the neonatal units such as providers’ characteristics, workplace factors, and group influences emerged as important elements possibly affecting the quality of neonatal care [16, 17]. Owing to their intrinsic nature and strict relationships with the personal providers’ experiences in the work setting, these features can be better represented by information from subjective judgments of care personnel rather than by formal objective evaluation with quantitative estimation. Two ideal types of work organization can be identified: a bureaucratic or task-centered model, and a professional or goal-oriented model. Because of the complex and somewhat unpredictable nature of working in an NICU, the professional model is preferable over the bureaucratic one. The term *nursing work environment* was proposed to define “the organizational characteristics of a work setting that facilitate or constrain professional nursing practice” [20].

In this study we investigated, using both quantitative and qualitative subjective estimators, some organizational and work environmental factors in a group of Italian NICUs adhering to the Italian Neonatal Network, branch of the Vermont Oxford Network. These NICUs represented half of all those present in Italy, and were evenly distributed among geographical areas in relation to the number of births per year in each area.

We found a very high variability in size of hospitals where the NICUs were located, in size of NICUs themselves and in volume of activity of each unit, expressed by the mean daily number of newborns hospitalized (a fourteen-fold difference between the lowest and the highest value!). We also found that nearly all the other performance indicators under study and variables in relation to the work environment showed ample variability among units. In particular, a nine-fold difference was present between the lowest and the highest value of the aNPR, indicating an inefficient distribution of human resources and possibly the presence of understaffed alongside overstaffed NICUs. This imbalance is confirmed by a more favorable aNPR in units with low volumes of activity compared to those with high volumes, a finding that implies higher nurse workloads in the largest NICUs.

The differences that emerged among geographical areas suggest the presence of a less flexible management of staff resources’ turnover (as documented by doctors’ and nurses’ mean age), less training possibilities, lower motivation, and worse qualitative evaluation of performances in Central and Southern compared to Northern regions. Small differences were also found in nursing work environment, while mean volumes of activity, characteristics of infants (as documented by the acuity score) and mean nurses’ provision were comparable. On the other hand, no differences were found in organization and work environment according to the mean daily number of patients.

In previous studies we observed a lower mortality for VLBW and very preterm infants in Northern compared to Southern Italian regions [5, 18]. Because the outcome of newborns admitted to NICUs participating in the present investigation was outside the remit of the study, we cannot know whether and to what extent the heterogeneity found among units (and in particular the differences that emerged among Northern, Central and Southern regions) are reflected in babies’ outcomes, although they are in themselves reasons for concern. Another aspect of our study requiring attention is that NICUs with low level of activity, estimated by the mean number of patients present per day, have a more favorable aNPR then large NICUs, indicating an uneven distribution of human and possibly also of financial resources among units [19].

The issues of nurse workload and staffing as important aspects of neonatal care were addressed in several papers [12, 15, 19, 25–27]. However, the various definitions and methodological approaches employed limit, up to this moment, the possibility to draw definitive conclusion about the relationship between workload and neonatal outcome [14]. Most studies, like our own, used the NPR adjusted for severity of patients as a measure of nurse workload, but, as Sherenian et al. pointed out, it is not clear how well such measure correlates with the quantity and intensity of a nurse’s duties, especially when other variables, such as fluctuating unit characteristics and the occupancy rate, are not taken into account [14]. Other important components of workload estimates are organizational factors, that can influence performances when intensity is already high, and the measurement of nurses’ own perception of their workload on a day-to-day basis [26]. It has however been recognized that methods of categorizing dependency have sometimes to give up precision in relation to individual babies in favor of ease of application in practice [27], especially in large scale studies.

The main limitation of this study resides on the fact that only half of the Italian NICUs adhered to the project, thus potentially restricting generalizability to the whole Country and other settings. Although the participating centers were evenly distributed among geographical areas in relation to the number of births in each area, we cannot rule out that some selection took place, in particular when considering that participation was voluntary. It is important, however, that in spite of a possible “positive” selection deriving from this kind of recruitment and of a consequent expected high homogeneity among neonatal units, actually we observed ample variability in results. On the other hand, having taken into consideration also a set of variables in relation to characteristics of hospitals, personnel and work environment, along with other more easily quantifiable and frequently employed variables such as the NPR adjusted for acuity of patients, represents the main strength of the study.

## Conclusion

In conclusion, we demonstrated the presence of high variability in some indicator of organizational characteristics and work environment among Italian NICUs and an uneven distribution of NPR adjusted for acuity of patients in relation to the volume of activity, suggesting that the larger the NICU the greater the workload for each nurse. We also found some differences in these indicators among geographical areas, in general with Northern regions performing better than Central and Southern ones. Although these results do not allow to identify reference standards, in particular for the NPR and the NICUs’ size, they can be useful to develop more expanded criteria for benchmarking and accreditation, including, along with usual outcome and procedural indicators, also variables associated with personnel characteristics and work environment. They should also prompt central and regional political rulers, administrative managers, and pediatric and neonatological medical societies to propose and implement urgent modifications in planning and organization of neonatal services in order to pursue more efficient, homogeneous and integrated regionalized care systems.

### Ethics, consent and permissions

Written consent to participate in the study was given by physicians and nurses in each NICU.

The study was performed in compliance with the Helsinki Declaration on medical research involving human subjects, and was approved by the Etchics Committee of Azienda Ospedaliera “Ospedale di Lecco” on March 04, 2009, ref. 140109. Local Ethics Committees’ approval was also sought by all units participating in the study.

## Abbreviations

VLBW: Very Low Birth Weight
NICU: Neonatal Intensive Care Unit
NPR: Nurse-to-Patient Ratio
aNPR: adjusted Nurse-to-Patient Ratio
AS: Acuity Score
